# Behind the wheel: community consultation informs adaptation of safe-transport program for older drivers

**DOI:** 10.1186/s13104-015-1745-0

**Published:** 2015-12-09

**Authors:** Kristy Coxon, Lisa Keay

**Affiliations:** Occupational Therapy, School of Science and Health, Western Sydney University, Penrith, NSW Australia; The George Institute for Global Health, Sydney Medical School, University of Sydney, Bridge Street, Sydney, NSW 2000 Australia; PO Box M201, Missenden Rd, Sydney, NSW 2050 Australia

**Keywords:** Driving, Education, Older drivers, Qualitative research, Safe transport

## Abstract

**Background:**

Safe-transport is important to well-being in later life but balancing safety and independence for older drivers can be challenging. While self-regulation is a promising tool to promote road safety, more research is required to optimise programs.

**Methods:**

Qualitative research was used to inform the choice and adaptation of a safe-transport education program for older drivers. Three focus groups were conducted with older drivers living in northwest Sydney to explore four key areas related to driving in later life including aged-based licensing, stopping or limiting driving, barriers to driving cessation and alternative modes of transportation. Data were analysed using content analysis.

**Results:**

Four categories emerged from the data; bad press for older drivers, COMPETENCE not age, call for fairness in licensing regulations, and hanging up the keys: It’s complicated! Two key issues being (1) older drivers wanted to drive for as long as possible but (2) were not prepared for driving cessation; guided the choice and adaption of the Knowledge Enhances Your Safety (KEYS) program. This program was adapted for the Australian context and focus group findings raised the need for practical solutions, including transport alternatives, to be added. Targeted messages were developed from the data using the Precaution Adoption Process Model (PAPM), allowing the education to be tailored to the individual’s stage of behaviour change.

**Conclusion:**

Adapting our program based on insights gained from community consultation should ensure the program is sensitive to the needs, skills and preferences of older drivers.

## Background

In motorised countries driving is commonplace in later life [1, 2]. While driving is beneficial [3–5] for independence and community participation, safety concerns for older drivers have been raised and supported by higher crash involvement per distance driven [6] and over-representation in intersection crashes involving multiple vehicles [2, 7, 8]. Considering the link between driving cessation and social isolation [9], depression [10–12] and even premature institutionalisation [12, 13], driving is not only important for independence, but overall well-being and quality of life. The challenge for policy makers and health educators is to develop licensing regulations and programs that strike an acceptable balance between safety and independence in community mobility for older drivers.

Some jurisdictions enforce age-based regulations for licensing in an attempt to revoke driving privileges of high-risk older drivers or restrict their driving to low-risk situations. In New South Wales(NSW) driver licensing regulations mandate annual medical assessments of fitness to drive from age 75 and choice of either on-road driving test biennially or radius restricted license from age 85 [14]. Although this policy context shares common ground with other jurisdictions across the United States(US) [15] and Canada [16], NSW has one of the strictest aged-based regulations in Australia and internationally. A recent review of the literature by Siren and Haustein [17] found no evidence for the benefits from age-based screening to outweigh the disadvantages. Further to this, one recent cross-sectional ecological study of mature driver laws across the US found having any restriction on licensing was actually associated with higher motor vehicle crash fatality rates for people over the age of 65 [18]. Further to this, regulations that deter or falsely label safe drivers as unsafe will cause premature loss of license with likely adverse outcomes for the person. Implementing age-based regulations that only detect unsafe drivers is important, but may be tricky.

Education is an alternative strategy that has been proposed to optimise safe driving practices among older drivers [19] through classroom modules [20], video programs [21], workbooks [5] and one-on-one counselling [22]. Self-regulation, by intentionally matching driving exposure to driving skills, is one practice that has been proposed to help older people drive safely for longer [23, 24] and is supported by epidemiological evidence linking self-regulation to declines in function [25–29], health status [30, 31] and poorer performance in on-road assessment [25]. Given this evidence, it is plausible that on-road safety can be enhanced through education to promote self-regulation for older drivers; however, there is a lack of trials evaluating the effect of such programs on safety outcomes [19].

Considerations of preferences, acceptability, accessibility and cost to older drivers are critical when developing strategies for safe mobility, but reviews of the literature have found a shortage of stakeholder perspective data from older drivers themselves [32]. Craig and colleagues [33] urge researchers to employ these perspectives in the development of complex interventions, especially prior to formal evaluation via trials. By understanding the perspective of the user, education programs are likely to be more effective, particularly when behaviour change is the desired outcome. For example, education may increase knowledge, but might not translate to behaviour change if issues such as preferences or availability of resources are ignored. To inform choice and adaptation of a safe-transport program for older drivers, focus groups were conducted to explore older drivers’ perspectives of age-based licensing, driving cessation and transport alternatives. In this paper we (1) present focus group findings, (2) compare findings to behaviour change stages of the Precaution Adoption Process Model [34, 35] to develop safe driving messages for community living older drivers and 3) describe adaptations made to an education program to support older drivers based on these findings.

### Program background

*Knowledge enhances your safety (KEYS) program* Although several programs promoting self-regulation among older drivers exist, the KEYS [22] program was ultimately selected as the base for our program. Originally intended for high-risk older drivers with visual deficits, the KEYS program was designed in the US to promote awareness of visual impairment and adoption of self-regulatory driving behaviours such as avoidance of high-risk driving situations [22]. The KEYS program involves two one-on-one sessions to counsel individuals in safe driving practices [22]. While not shown to protect against crash risk [36], this program was well grounded in Social Cognitive Theory [22] and has been shown to increase safe driving practices among high-risk drivers with visual impairment [37].

*The precaution adoption process model (PAPM)* Craig and colleagues [33] suggest complex interventions may work better if there is an agreed level of flexibility in the protocol. To customise our education, but preserve program fidelity, we standardised the program’s underlying behavioural change theory. The Precaution Adoption Process Model (PAPM) [34, 35] was used to determine stage of precaution adoption for each older driver allowing the educator to tailor the education to meet the needs of the driver. Utility of the PAPM framework has been shown in previous studies of health-behaviour adoption [34, 38–40] and paediatric injury prevention [41]. It has also been used to explore the process of driving cessation and self-regulation among older drivers [42].

The PAPM is a stage-based model of behaviour change that focuses on both hazard and precaution [34, 35], in our case, crash/loss of driving privileges and adoption of self-regulatory driving practices. The process of change is characterised by seven stages including stage (1) unaware of issue, stage (2) aware of issue but unengaged, stage (3) deciding about action, stage (4) decided not to act, stage (5) decided to act, stage (6) acting and stage (7) maintenance [43]. The PAPM makes a unique distinction between people who are not aware versus people who are aware but not personally engaged with the issue. As different messages are required at varying stages of precaution adoption to move the person to the next stage towards sustained action [34], this distinction, along with inclusion of a stage dedicated to those who decided not to act, allows educators to tailor the education to meet the behaviour change needs of the person. For example, people who are unaware of an issue (stage 1) need information to raise their awareness of the issue and precaution, while people who have decided to act need information on how to implement the behaviour (stage 5). ‘Hard to reach’ people who have decided not to act (stage 4), need targeted messages to tip the risk–benefit ratio toward adoption of the behaviour [43].

## Methods

### Data collection

Focus groups were employed to explore four key areas; aged-based licensing, stopping or limiting driving, barriers to driving cessation and alternative modes of transportation. Group interaction was considered valuable, providing a safe environment among peers for generating ideas, sharing and validating experiences [44, 45]. As previous research indicates differences in the way men and women relate to driving [46–49], focus groups were split by gender to enhance homogeneity within groups and preserve latent gender differences in driving. Each group was led by an experienced researcher (LK or ML) trained in qualitative research methods. To ensure consistency between groups, a semi-structured discussion guide of open-ended questions to elicit data on key topics was used by each facilitator to steer the discussion (Table 1). Focus groups were conducted in a local community centre, familiar to participants, to enhance comfort and promote open discussion [45]. Focus groups lasted between one and 2 h, were audio-recorded and transcribed verbatim. Data were managed in NVivo10 (QSR International Pty Ltd). Due to the nature of the group setting, it was not possible to accurately identify or link data from individual participants in the transcript. As focus group methodology aims to develop a collective understanding of the phenomenon under investigation [45], identifying each participant’s contributions was not considered essential.

**Table 1:** Facilitator guide used to direct focus group discussions

Discussion topics	Probes and questions
NSW licensing and age	Explore awareness of regulations e.g. Tell me what you know about the licensing regulations for older drivers in NSW? Explore opinions of current regulations—including equity, implications for older drivers e.g. What do you think about those regulations?
Stopping or limiting driving	Under what circumstances would you not drive? Are there situations when you do not drive or do not feel confident driving? How do you make the decision to not drive? Do you get advice?
Barriers to stopping driving	Are there situations where you have to drive? Tell me about those situations What are the implications of not driving?
Alternate modes of transportation	Explore familiarity of alternate transport including public transport, taxi schemes, community buses e.g. What alternatives are available? What alternatives do you use? What alternatives would you use? What is your preferred scheme? How useful is that scheme? Explore acceptability of cost of alternate transport schemes

### Participants

Older drivers were recruited through advertisement in local newspapers and seniors’ groups in a semi-rural area approximately 35 km north-west of Sydney. People interested in participating could register their interest via the study office or sign-up sheet at various seniors’ groups. Eligible participants held a current drivers’ license (full or restricted) and spoke conversational English. As there is no hard and fast timeframe for decline in driving skills as people age, no age limit was set to capture a variety of issues and experiences related to driving in later life. Anyone who considered themself an ‘older driver’ was eligible to participate. The study had approval from the University of Sydney Human Research Ethics Committee and written consent was obtained from each participant.

Three focus groups, one male and two female, were conducted in November 2009. Each focus group had between four and six participants. All participants were members of community-based seniors clubs in north-west Sydney. To join these groups members are required to be in the retirement phase of their life (semi or completely retired from work). A total of 15 participants took part, 5 males and 10 females. Age and other demographic information were not recorded.

### Context

Licensing requirements for older drivers vary between states in Australia. All participants lived in NSW and were subject to the licensing regulations of this jurisdiction.

### Analysis

Content analysis using inductive methods was used to analyse the data [50]. To check preliminary understanding of the data, a summary of each discussion was reviewed with participants at the end of each focus group to clarify and confirm our interpretation. A conventional approach, described by Hsieh and Shannon [51], was taken to allow codes and categories to emerge from the data. Two researchers were involved in this analysis (LK and KC). Both researchers immersed themselves in the data by listening to recordings and reading transcripts. Codes were generated separately but sequentially by each researcher. The initial coding scheme from the first analysis (LK) was used as the framework for the second analysis (KC). Through a process of discussion and comparison, author consensus was achieved and codes sorted into categories [51].

### Program adaptation

Focus group findings were used to inform choice and adaption of the program. Categories were compared to each stage of the PAPM [34]. These findings were used to tailor educational messages to meet the behaviour change needs of each stage.

## Results

### Categories

Content analysis revealed four categories; bad press for older drivers, COMPETENCE not age, call for fairness in licensing regulations, and hanging up the keys: it’s complicated! (Table 2). Each category and *subcategory* is described below.

**Table 2:** Categories and subcategories emerging from focus group findings

Categories	Sub categories
Bad press for older drivers	Misconceptions about older drivers Prejudice
Competence not age	Experience counts Older is wiser Driving skills can change over time
Call for fairness in licensing regulations	Falling through the gaps of age-based testing The doctor debate The on-road test ‘We should all be tested’
Hanging up the keys: it’s complicated	What’s the alternative? Car equals convenience Car is identity Self-regulation is the compromise Knowing when to stop driving

#### Category 1: bad press for older drivers

Participants felt that older drivers receive bad press. Although it was acknowledged that safety concerns exist for a minority of older drivers, participants felt incidents involving older drivers were not well represented in the media, and overestimated by the driving public. Participants argued there are larger safety issues with young, inexperienced and aggressive drivers.

‘There is a higher accident rate for the up to 25 year olds than there is amongst the over 75 year olds and yet they’re saying, they pick on the individual accidents and things we have—they say “Oh there you are, see I told you so, these over 80 blokes, they don’t know what they are doing!”’

Bad press was seen to feed *misconceptions* such as old is slow;

‘“Don’t get behind X [name] because you’ll never get there! He’s too slow.” And I got that from all the locals…’

as well as *prejudice* towards older drivers. With alarm, participants described on-road experiences where they felt intimidated by other drivers who overtook them or drove dangerously ‘just to scare’ or teach them a ‘lesson’. The lesson being ‘keep off the road!’ For example;

‘And I was coming down [this] road… and a bloke came up the other way, crossed over to my side of the road, drove at about that far off me and then back onto his side of the road again. Just to scare me.’

#### Category 2: COMPETENCE not age

There was consensus that licensing regulations should be based on competency not age;

‘But one of the things all of us, I think, agree with, is our competence to drive is not related to our age.’

Participants felt strongly about this and supported their claim with examples of successful older drivers such as;

‘I’ve known 90 years olds who – I remember X[name], 92, 93, could drive bright as a button, so age is not really the thing, it is your competence to drive’ and ‘I heard cheers in the church one morning when he said he passed his driving test and he was 90…’

Participants discussed driving incidents and crashes involving other drivers, particularly younger drivers, to highlight there are bad, if not worse, drivers in other groups.

Participants presented their case for competence explaining *experience counts* and being *older is wiser* behind the wheel. Participants reflected on their past driving practices with heightened understanding of the costs and benefits they suggest comes with age and experience. Most reported abandoning past unsafe practices, opting for safer, more conservative, driving practices in later life;

‘At one stage I remember driving from here to up near Ballina [832kms], I think it was a tank full of petrol and doing a fair speed… I left at 4 o’clock in the morning and got up here at about 5 or 6 o’clock at night…I’m talking about when I was in my 40 s…now that I am getting towards double that age, I look back and say how stupid! And what did I do with that time that I saved?… All you did was walk around feeling a bit of a zombie and worn out.’

With a slower pace commonly experienced in retirement, participants saw no need to speed;

‘Well what’s the point of driving at that speed if you’ve got plenty of time to get there?’

while also recognising the futility of speeding;

‘They want to gain an extra two minutes by speeding!’ and

‘If you can get in a Ferrari and you can do nought to 100 in five seconds and my Holden… will go from nought to 100 in 10 s. All the question is, what are you going to do with the five seconds you saved?’

Despite the safety benefits that come with age and experience, there was acknowledgement that *drivingskills can change overtime*;

‘Like my father, it was just little minor things, we realised his judgement obviously wasn’t as good.’

However, participants argued that we are all different and changes can occur at any age;

‘Doesn’t matter whether you’re 55, you can’t drive if you’ve got a white stick. It’s competence, not age.’

#### Category 3: call for fairness in licensing regulations

The assertion that driving competency is not necessarily related to age prompted a call for fairness in licensing regulations. Most were not opposed to the concept of age-based testing, stating ‘you should have the test’ and ‘they will always have some sort of criteria for when they start.’ However, although a minority opinion, some participants felt older drivers were ‘over-governed’ with ‘too much bureaucracy.’ Nevertheless, participants agreed that vision loss, declining cognitive function and medical conditions were valid reasons for being required to stop driving.

In terms of fairness, several problems with current licensing regulations emerged from the data. Firstly, some drivers were *falling through the gaps of age*-*based testing*. Some drivers with deficits were not correctly identified or stopped from driving through the licensing system. For example;

‘We sort of insisted that he [Doctor] not approve Dad…he wouldn’t have really have known how Dad’s driving was. He used to arrive in the surgery, he was fine, but his driving by this time was a bit erratic, anyone behind him was always saying Dad shouldn’t be driving!’

The need for intervention by a third party to ensure an appropriate licensing outcome was echoed in other examples;

‘I took my husband to the doctor and asked if he could be stopped…he has peripheral neuropathy and could not tell whether his foot was on the accelerator or the brake’ and

‘When I was working with all of these doctors, it was the relatives who came and asked, never the person.’

Participants stressed that the current system was open to corruption through practices such as doctor shopping, whereby if a doctor refused to sign a medical fitness to drive certificate, the older driver would find another doctor who would sign it;

‘Of course my Dad dropped him and went to another doctor!’

Drivers with deficits may fall through these gaps in the system that allow drivers to ‘shop’ around for medical clearance.

The second problem identified was the question of whether the general practitioner (GP) was the right person to assess fitness to drive or not. On one side of *the doctor debate,* the doctor was deemed to be appropriate;

‘If you have an underlying health problem which would preclude you from driving, your doctor would have to know about it.’

In particular, GPs may be vital in detecting dementia or changes in cognition over time. On the other side, there were concerns that assessments vary between practitioners, a medical check ‘doesn’t say how you drive’, and unless the GP specifically asks, they do not necessarily know if people are still driving. There was acknowledgement that there are a ‘lot of people and a lot of pressures on a GP,’ and concern that GPs working in medical centres may not know the patient well and therefore need to rely on self-report and medical notes;

‘It’s what the person tells them, the doctor is only as good as what they are telling him.’

Thirdly, concerns about *theon*-*road test* emerged. Participants who had taken the test felt the outcome was dependant on the ‘mercy’ of the driving assessor, questioning reliability between assessors. Human factors such as anxiety also influenced performance on the test;

‘It is the closest I have been to having a nervous break-down, it was time to take the test!’

While others spoke of their future fear of taking the test;

‘I would be frightened of losing my license.’

Although there was clear support for correctly detecting drivers with deficits, and revoking or restricting driving privileges accordingly, there was a strong belief that this should be across all drivers of all ages. Participants saw transparency as key to a fair, just and accurate system, that is, *‘We should all be tested!’*

#### Category 4: hanging up the keys: it’s complicated!

Making the decision to stop driving was seen as ‘a very difficult decision,’ complicated by a myriad of factors. These factors may facilitate, hinder or even override timely decisions to cease driving.

Firstly, a lack of alternative transport was seen as a major barrier to driving cessation, leaving participants asking the question; *What’s the alternative?* Community transport was seen as a good option but limited to medical appointments. The problem with this is ‘people like to go shopping; they like to get their hair done’, therefore, community transport for medical appointments does not meet individual transport needs. Bus services in the area are scarce, trains non-existent and taxis expensive, particularly for semi-rural residents. Many suggested they would be reliant on family and/or friends for transport but could not ‘expect it.’ Several participants concluded moving to a place with plenty of public transport would be the only option;

‘If I had to give up driving the first thing that would have to happen is I would have to sell out. I would have no choice.’

However, this was not ideal as moving suburb means leaving behind established social networks that are important in later life;

‘And then of course, you get all sorts of problems of being taken out of the place where you have the relationships and friendships…I’ve seen it again and again and again…they’ve given all their relationships away down there, and here they’re strangers and they have to redo it all again at the time when they’re least able to do it.’

The next factor was the convenience of a car. A *car equals convenience,* and convenience can be difficult to forego. Despite the lack of alternative transport, there was strong personal preference to drive, and driving for as long as possible was the ultimate goal. Driving means you can go where you want, when you want, which makes the decision to stop even harder, particularly given the lack of appropriate alternatives. For example;

‘To see his daughter by bus-you’ve got to walk to a bus-stop where you know there is a bus which takes you to some place where you’ve got to change into another bus that takes you to get into another bus or train to get to your daughters. You see? With your car, you could be there in 20 min. See, cars are so convenient for us at our age, just to get in and go somewhere without any worries.’

There are also trips that would be impractical on public transport, such as;

‘I couldn’t put my golf clubs on a bus. How would I play golf?’

Another factor to consider is a car means more than independence to these drivers, a *car is identity*;

‘The car is everything- it is who they are. It has to do with status and personality.’

Driving a car was closely linked to a person’s roles and responsibilities, who they are and what they do. This complicates any decision to stop, particularly the responsibility to drive others such as a non-driving spouse or grandchildren;

‘My wife looks like losing her eyesight, it is on the bend now, she has macular degeneration and if she loses her license then we will be totally dependent on me having mine. So you get complication.’

Participants described a transition to not driving where *self*-*regulation is the compromise*. Strategies such as avoiding night driving, peak-hour traffic, and adverse weather conditions (heavy rain, fog), were being employed by many participants as well as their peers;

‘I think a lot of people do, you talk to people and they say “I don’t drive at night, I only go out in the daytime” and that’s not unusual.’

Participants described several different reasons for changing their driving exposure including response to age-related changes (particularly vision), avoiding road congestion, no longer feeling comfortable driving in certain circumstances as well as simply not needing to go to a certain place or out at night. Although this driving restriction was generally seen as a way to stay driving for longer, participants cautioned if a person doesn’t drive as much, they are at greater risk of losing their driving skills and confidence;

‘With reservation that as your restrict the distance you drive, that you become more cautious about it’ and

‘It is unfortunate, when you haven’t driven much for a year; it is less and less likely that I will take the test again.’

Although self-regulation involved some planning, there was a distinct lack of planning for driving cessation. There were many future transport problems raised, without planned solutions for continued community participation beyond driving. Driving cessation was seen as incompatible with continued residence in this area due to lack of available alternatives.

The final factor was about *knowing when to stop driving*. Most were confident that driving cessation would be self-directed and they would know when it was time to stop; ‘I would just stop when I thought it was time,’ but were less concrete about how they would know. For example;

‘I think when I start getting frightened of my own judgement. I didn’t see that car, or I didn’t see the lights or gee, I didn’t stop quick enough. Then I’ll think to myself, there’s something wrong with me. If this is going to keep happening, I’ve got to give it away.’

Many thought they would be ‘good’ judges about when they would need to stop, while others felt they would need external advice from family or practitioner:

‘I think for myself I probably wouldn’t know when not to drive and it would probably be one of my children that would say “Come on Mum, you are passed it.”’

### Program choice and adaptation

These findings were used to inform choice and adaptation of our safe-transport program aiming to assist older drivers make safe and informed transport decisions while maintaining their community mobility. Two key issues emerged from the data; (1) older drivers wanted to drive for as long as possible but (2) were not prepared for their transition to not driving. Given these findings along with support from epidemiological research, self-regulation was chosen as the central strategy for the program, and the KEYS curriculum nominated as the framework for delivery and content.

**Fig. 1:**
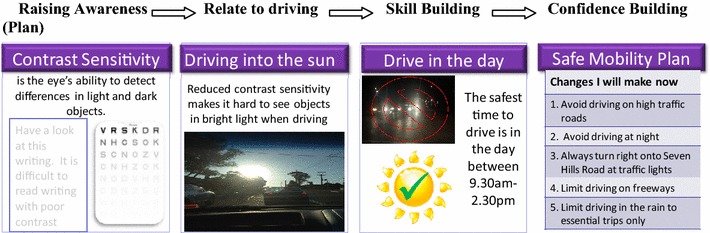
Example from adapted safe-transport program for older drivers named ‘Behind the Wheel’

To address the key issues identified from our data, we adopted two educational goals; (1) to assist older people to drive safely for as long as possible while (2) preparing them for retirement from driving. Adaptations were made to the KEYS curriculum to achieve these goals. Being a US-based program, we needed to substantially change content to make it relevant to the Australian driving context. Manual images were replaced with photographs of local driving environments, language changed to reflect Australian terms (e.g. peak-hour replaced rush-hour) and self-regulation strategies adapted to account for driving on the left-hand side of the road. The KEYS program was developed for older drivers with impaired vision. To target drivers aged 75 years and over, without specific impairment, we extended content to raise awareness of physical, cognitive as well as visual changes that may affect driving in later life. Functional assessments were administered and discussed with participants to raise their awareness of their skills and abilities. This information provided a platform to help drivers match their driving exposure to their skills; allowing the older driver to build skills in translating this information into appropriate self-regulatory strategies to extend safe driving for as long as possible (Fig. 1). Avoiding or limiting driving in the Australian equivalent of seven high-risk driving situations identified in the KEYS program (driving at night, in the rain, right-hand turns across oncoming traffic, heavy traffic, driving on high-speed highways and freeways, in peak-hour and driving alone) [22] along with driving in low speed school zones during their hours of operation were included as possible self-regulatory strategies in our program.

**Fig. 2:**
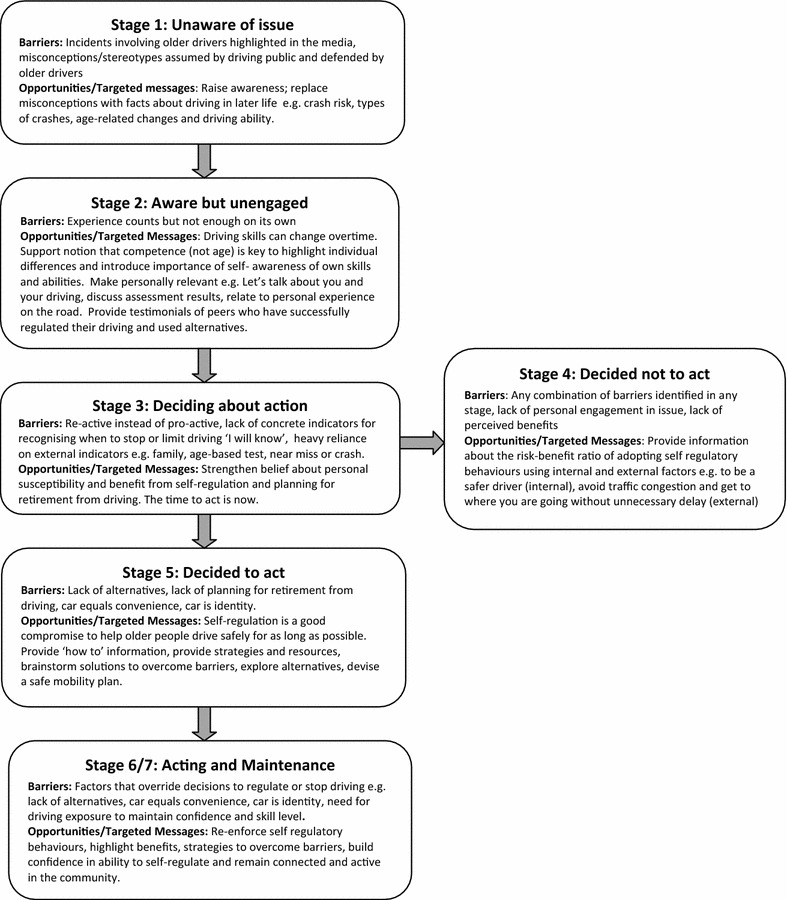
Tailored PAPM framework messages derived from older driver focus group findings

Recognising education programs need to be responsive to individual needs, standardised flexibility, using the participant’s PAPM stage, was introduced. Our qualitative research findings were used to generate stage-based targeted messages that address issues relevant to older drivers. Figure 2 shows how these findings informed educational messages to target older drivers at each stage of the PAPM. To allow the educator to deliver this tailored education to each older driver, our program adopted the unique one-on-one format used by the KEYS program [22] for delivery of two sessions. We chose to deliver the education in participants’ homes to facilitate feelings of comfort and control over the process, in the hope that this sense of control would extend to ownership over the behaviour change process.

To address lack of preparedness for driving cessation, we used an occupational therapy problem solving process [52] to help older drivers consider life after driving cessation. Using both the PAPM stage to target messages and the educational strategies of raising awareness, building skills and gaining confidence employed by the KEYS program [22], we prepared a package focusing on helping older drivers plan for retirement from driving to stay active, and connected to their community. To formulate this plan, inventories of usual trips were taken to allow the educator to match available alternative transport in the local area to desired trips. Participants were encouraged to brainstorm personally relevant and preferred alternatives, and start using these alternatives to familiarise, develop skills and gain confidence in their use. By helping older drivers become proactive, not reactive, in their planning for driving cessation, we aimed to promote the likelihood of smooth and successful transitions to not driving at the appropriate time.

## Discussion

Exploring the perspectives of older drivers was an important first step in understanding the issues, challenges and needs of older drivers in the community and essential to the adaptation of a safe mobility program for older drivers. For these participants, driving was key to staying mobile in later life, particularly given sparse transport alternatives in semi-rural areas. To preserve integrity of driving privileges, participants called for fairness in licensing regulations with competence, not age-based testing, recommended to detect unsafe drivers of all ages. The decision to hang up the keys was complicated by a myriad of competing factors, many of which override the need to stop or limit driving, highlighting the challenges older drivers face when contemplating driving cessation. Despite these issues, few participants had planned for a time when driving was no longer possible, leaving uncertainty about future transport options and community mobility.

Self-regulation, such as avoiding night driving and peak-hour traffic, was described by participants as a way to drive safely for longer; which supports self-regulation as the central strategy for education. Finding a compromise between unrestricted driving and driving cessation when function starts to decline was important to participants; however, strategies to enhance this were not raised. Previous research suggests other reasons [42, 53], such as no longer needing to venture out at night, also contribute to changed driving patterns among older drivers, and our data supports this. Although changes to driving exposure may not always be self-regulation [54] as intent cannot be confirmed, population-based surveys in the US and Australia confirm one quarter to one-third of older drivers made at least one adaptation to their driving [25, 55, 56]. Regardless of the reason for change, educational programs that enhance self-regulation or avoidance of high-risk driving situations have intuitive merit, are supported by epidemiological data associating self-regulation with reduced crash risk and warrant further evaluation to determine safety and community participation benefit. Such strategies also have potential to preserve both convenience and identity associated with driving a car; two important factors participants felt complicated the decision to cease driving.

Two key issues emerged from the data; (1) older drivers wanted to drive for as long as possible but (2) were not prepared for their transition to not driving. These messages were congruent with previous research that found driving for as long as possible was important to older drivers for both practical [1, 42, 57] and symbolic reasons [1, 42, 57], however, older drivers were largely unprepared for driving cessation [1, 42, 57–59]. This lack of preparedness for driving cessation has prompted several authors to call for comprehensive programs to help older drivers recognise the appropriate time to cease driving and plan in advance for this transition [5, 46, 59, 60]. These findings, along with evidence that planning helps ameliorate negative effects associated with driving cessation [46, 59], supports the data-driven educational goals assigned to our program. Working toward both goals simultaneously (driving as long as possible while planning for driving cessation) is supported by evidence that suggests the optimal time to prepare for retirement from driving to achieve successful transition is while still driving [1, 46, 57, 60].

Hassan and colleagues [42] found the PAPM to be a useful framework for understanding self-regulation among older drivers. In our research we took the use of the PAPM a step further, by using the PAPM as a framework to target educational messages to promote self-regulation among older drivers. Stage-based messages, informed by our qualitative data, were generated using the PAPM framework (Fig. 2). Based on educational needs unique to each PAPM stage [43] and our findings, these messages were designed to address barriers relevant to older drivers in order to move them toward sustained adoption of self-regulatory driving practices. Echoing earlier research findings [5, 42], our data revealed lack of transport alternatives as a major barrier to driving cessation. Developing stage-based messages allows the educator to address both hazard and precaution/solution by framing the message, in this case, planning for retirement from driving, to meet the behaviour change needs of the older driver. For example, using testimonials to engage older drivers with alternatives in stage 2, introducing benefits of planning for driving cessation now in stage 3 versus providing ‘how to’ information on accessing alternatives in stage 5. A randomised controlled trial to evaluate behaviour change among older drivers using these educational messages is underway [61].

Like all studies, several limitations need to be considered when interpreting the findings of this study. Firstly, it was not possible to accurately link participant’s contributions as the group setting made it difficult to identify specific participants in the transcript. Secondly, age and demographic information was not recorded, so we are unable to report the age-range of participants. Finally, all participants resided in the semi-rural outskirts of northwest Sydney. Driving exposure among older drivers can vary in semi-rural settings [62] where public transport is scarce which may change driving decisions in later life. Participants were likely to have similar levels of education and socio-economic status, however, the homogeneous nature of the sample was apt to inform the adaptation of a safe-transport program intended for older drivers in this area. Although this data was collected in 2009, there have been no major changes in demographics, transportation or age-based licensing requirements for older drivers living in this region. We therefore, feel that the emergent categories are still relevant to older drivers living in northwest Sydney today.

## Conclusion

Consultation with older drivers in the community provided valuable insights to adapt an education-based program to promote safety but maintain mobility. Retiring from driving was considered a major life decision, subject to a large number of considerations. Self-regulation appealed but current policies were criticised as ageist and flawed. Though the program content was based on an existing program, several adaptations were necessary for the Australian context and the process of tailoring the content to the stage of engagement in the process of retiring from driving. The focus groups highlighted the need for alternatives and practical solutions and these were added to the program. Incorporating the perspective of older drivers in the design of our program should ensure the program is sensitive to the needs, skills and preferences of the older driver.

## Abbreviations

KEYS: knowledge enhances your safety
PAPM: precaution adoption process model
NSW: New South Wales
US: United States
GP: general practitioner
